# Author Reply: In Response to “De Novo *KRAS* G12C-Mutant SCLC: A Case Report”

**DOI:** 10.1016/j.jtocrr.2022.100425

**Published:** 2022-10-19

**Authors:** Meridith L. Balbach, Rosana Eisenberg, Wade T. Iams

**Affiliations:** Division of Hematology-Oncology, Department of Medicine, Vanderbilt University Medical Center, Nashville, Tennessee; Vanderbilt University School of Medicine, Nashville, Tennessee; Vanderbilt-Ingram Cancer Center, Nashville, Tennessee; Department of Pathology, Microbiology and Immunology, Vanderbilt University Medical Center, Nashville, Tennessee; Division of Hematology-Oncology, Department of Medicine, Vanderbilt University Medical Center, Nashville, Tennessee; Vanderbilt-Ingram Cancer Center, Nashville, Tennessee

To the Editor:

We thank Moore et al.^1^ for their thoughtful consideration of our patient case with de novo *KRAS* G12C-mutant SCLC. We agree that the variant allele frequency, although quite high in this patient, does not ensure the importance of this mutation in driving the patient’s SCLC. In the era of ever-increasing breadth and depth of tumor genomic profiling, a plentitude of patients with “targetable” mutations in noncanonical settings continue to present challenges and opportunities akin to this case. The ability to reliably estimate the cancer effect size of individual mutations is a tool of immense value to molecular tumor board discussions, clinical trial design, and practicing oncologists. To fully realize this potential, we believe that the next step is acquiring clinical validation data incorporating the prospective use of cancer effect size quantification (and other systems biology models) to guide clinical trial design and therapy selection.^2^ Such an innovative approach expands on the aims of the NCI-MATCH (Molecular Analysis for Therapy Choice) trial, providing an evolutionary and systems biology perspective to guide treatment selection beyond simply identification of single-gene alterations or individual protein expression levels.^3^^,^^4^

We are hopeful that through a concerted effort among our cancer biology experts, clinical trialists, and the NCI, we can continue to advance the rigor of scientific rationale guiding the deployment of our ever-expanding cancer therapy repertoire. For more information on such work and to further engage, please consider participating in the upcoming NCI Symposium in Spring 2023 on the implementation of systems biology models to clinical trials. In collaboration with Dr. Andrea Bild at the City of Hope, we hope to engage multiple collaborators in our WE-MUST (Wielding Evidence in a Multicenter Umbrella Systems Trial) approach.

## CRediT Authorship Contribution Statement

**Meridith L. Balbach:** Conceptualization, Writing—original draft, Visualization, Project administration.

**Rosanna Eisenberg:** Resources, Investigation.

**Wade T. Iams:** Conceptualization, Resources, Writing—review and editing, Supervision.
